# Evaluation of analytical performance of Alinity i system on 31 measurands

**DOI:** 10.1016/j.plabm.2020.e00185

**Published:** 2020-10-20

**Authors:** Jong Do Seo, Da Young Song, Youngwon Nam, Chihchiao Li, Seunghwan Kim, Joon Hee Lee, Kyunghoon Lee, Junghan Song, Sang Hoon Song

**Affiliations:** a Department of Laboratory Medicine, Seoul National University Hospital, Seoul, South Korea; b Department of Laboratory Medicine, Seoul National University College of Medicine, Seoul, South Korea; c Department of Laboratory Medicine^3^, Seoul National University Bundang Hospital, Seongnam, South Korea

**Keywords:** Alinity i system, Analytical performance evaluation, Immunoassay

## Abstract

**Introduction:**

Accurate, precise and reliable laboratory test results play a critical role in medical decision making. To satisfy the increasing needs in clinical laboratory tests, the analyzers have been advanced. In this study, authors aimed to evaluate the analytical performance of the Alinity i system (Abbott Laboratories, IL, USA) for diverse analytes measured by using immunoassay principle.

**Materials and methods:**

Analytical performance of recently launched Alinity i system has been evaluated for 31 assays in aspects of precision, linearity and analytical measurement range, correlation with the Architect i2000sr system (Abbott Laboratories), carry-over, and reference interval validation in accordance with CLSI guidelines.

**Results:**

The within-laboratory CVs of the analytes tested in the study ranged between 1.00 and 7.84%, which met vendor claimed value in precision. In linearity test, most assays satisfied acceptable linearity criteria, best-fit first order regression or polynomial regression with nonlinearity smaller than ±10%, compared with linear regression. The recovery of each analyte distributed from 90.1 to 109.7%. The coefficient of determination (R^2^) for each test was larger than 0.95 except for folate when compared to the results obtained from existing routine analyzer and statistically or clinically equivalent. The carry-over rates were acceptable, and reference intervals were validated.

**Conclusion:**

Through this study, acceptable analytical performance of novel Alinity i system has been verified. It is expected to readily replace existing instrument and to be an option for laboratories considering introduction of automated immunoassay analyzer.

## Highlights

• The analytical performance of Abbott Alinity i system, recently launched by Abbott Laboratories, was evaluated.
• The analyzer was revealed to show reasonable precision, linearity, and carry-over.
• The platform showed good correlation with Architect i2000sr system, therefore readily replace existing instrument.

## 1 Introduction

With the recent medical advancements, many novel tests for diagnosing disease and determining therapeutic response have been developed and introduced in clinical practice. In order to meet increasing need in the clinical laboratory test, the number of assays performed and the samples handled in the laboratory are increasing [1]. Laboratory test results play a critical role in the screening and diagnosis of disease, determination of treatment strategies, evaluation of therapeutic responses, and clinical studies [2,3]. Therefore, accurate, precise and reliable laboratory test results are essential for optimal medical decision making [4].

The automated analyzer used in the laboratory have been advanced to reduce the turn-around time, have high throughput, and have improved accuracy to satisfy clinical demands. In addition, it has evolved by automating more processes to reduce labor and utilizing laboratory space through compact equipment design [5]. As the clinical chemistry section is a big part of the laboratory with accompanying automation, introduction of the new analyzing instrument is more active than any other section in laboratory.

The Alinity i system which has been developed by Abbott Laboratories, Abbott Park, IL, USA, has been developed to maximize the throughput and efficiency through compact and scalable design. There are some publications on the Alinity hq system, the hematology analyzer [6] or on the Alinity c system for chemistry [7]. However, there are few publications on the analytical performance evaluation of the Alinity system for immunoassay tests helpful when considering introduction of the instrument into a clinical laboratory. In this study, we aimed to evaluate the analytical performance of Alinity i system for diverse immunoassays.

## 2 Materials and methods

The Alinity i system (Abbott Laboratories, IL, USA), a recently launched automated immunoassay analyzer utilizes chemiluminescent microparticle immunoassay (CMIA) principle, by using anti-analyte coated paramagnetic microparticles and anti-analyte acridinium-labeled conjugates. The reaction is measured as relative light units, which have a direct or inverse relationship with the amounts of analyte in the sample [8].

Analytical performance of the system was evaluated for thirty-one assays, thyroid stimulating hormone (TSH), total triiodothyrionine (TT3), free triiodothyrionine (FT3), total thyroxine (TT4), free thyroxine (FT4), thyroid peroxidase antibody (anti-TPO), alpha-fetoprotein (AFP), carcinoembryonic antigen (CEA), carbohydrate antigen 19–9 (CA 19–9), cancer antigen 125 (CA 125), cancer antigen 15–3 (CA 15–3), human epididymis protein 4 (HE 4), total prostate specific antigen (TPSA), free prostate specific antigen (FPSA), testosterone, progesterone, estradiol (E2), prolactin, beta-human chorionic gonadotropin (b-HCG), follicle stimulating hormone (FSH), luteinizing hormone (LH), cortisol, high-sensitivity troponin I (hsTnI), brain natriuretic peptide (BNP), ferritin, intact parathyroid hormone (iPTH), folate, vitamin B12, 25-hydroxy [OH] vitamin D, C-peptide, and homocysteine, in aspects of precision, linearity, analytical measurement range (AMR), and carry-over rate as well as correlation with the Architect i2000sr system (Abbott Laboratories).

This study was approved by the Institutional Review Board of Seoul National University Hospital (1810-080-980), and informed consent was waived as residual patient samples was utilized and personal information was excluded in this study.

### 2.1 Evaluation of analytical performance

#### 2.1.1 Precision

The precision of the system was evaluated according to Clinical & Laboratory Standards Institute (CLSI) EP15-A3 guidelines [9], five replicates per run and a single run for five days. The control materials from Multichem IA Plus (Technopath Clinical Diagnostics, Ballina, IRL) were tested for cortisol, LH, and TT3. Abbott control materials were tested for the other assays. Depending on availability of control materials, two- or three-concentration levels of the controls were evaluated. Mean concentration, within-run precision, within-laboratory precision, coefficient of variation (%CV) and 95% confidence interval (CI) were calculated for each assay.

#### 2.1.2 Linearity and AMR

In accordance with CLSI EP 06-A guideline [10], evaluation of linearity and AMR were carried out by using commercialized linearity materials, Validate linearity sets (Maine Standards Company, ME, USA) for TT3, FT3, FT4, CEA, CA 19–9, TPSA, FPSA, testosterone, progesterone, prolactin, FSH, cortisol, ferritin, folate, and vitamin B12. For assays without available commercial linearity materials or uncovered by the manufacturer’s claimed AMR, either Abbott calibrators or patient serum samples were utilized for TT4, CA 125, HE 4, b-HCG, LH, hsTnI, BNP, iPTH, and 25-OH vitamin D, and for TSH, anti-TPO, AFP, CA 15–3, E2, C-peptide, and homocysteine, respectively. Every test materials were prepared to have five-concentration levels, then measured mean of quadruplicate measurements was calculated and compared with the expected value to yield recovery. When polynomial regression analysis for first-, second- and third-order polynomials did not show significant nonlinear coefficient, the test was regarded as statically “linear” in the measured range. When the best-fit regression was nonlinear, the recovery at each point was calculated, and difference between linear regression and best-fit regression were compared to obtain nonlinearity at each level. Tests with recovery distributed within range of 90–110%, or nonlinearity smaller than 10% for all level were regarded as clinically “linear”.

#### 2.1.3 Method comparison

Method comparison studies were performed using residual patient serum samples, based on CLSI EP09-A3 guideline [11]. The Alinity i system was compared with the Architect i2000sr (Abbott Laboratories). The samples were selected to cover AMR as wide as possible, more than fifty samples were collected for each assay. Deming regression was used for analysis to calculate a slope, an intercept and 95% CIs. The results were regarded as comparable without significant bias if the coefficient of determination (R^2^) was larger than 0.95, and the 95% CI for slope and intercept include 1 and 0, respectively. When the comparison results were not met by these criteria, an estimated mean percent bias was compared to the total allowable error (TEa) provided by the Westgard desirable biological variation database [12] to assess clinical significance of the bias.

#### 2.1.4 Carry-over rate

Serial measurements of high- and low-concentration materials (H1–H2–H3–H4-L1-L2-L3-L4) were carried out and carry-over rates were calculated according to the formula below. Carry-over rates smaller than 1% were considered acceptable.

Carry-over (%) ​= ​[L1-(L3+L4)/2]/[(H2+H3)/2-(L3+L4)/2)]∗100

#### 2.1.5 Reference interval validation

In accordance with CLSI EP28-A3 guideline [13], the manufacturer’s claimed reference intervals for TSH, TT3, FT3, TT4, FT4, anti-TPO, AFP, CEA, CA 19–9, CA 125, CA 15–3, TPSA, testosterone, prolactin, b-HCG, hsTnI, BNP, ferritin, iPTH, folate, vitamin B12, C-peptide, and homocysteine were validated using residual patient samples. Whole blood samples in ethylenediaminetetraaceticacid (EDTA) from individuals undergone routine health check-up were collected for BNP assay that mandates EDTA plasma as a reference specimen, and residual serum samples from individuals undergone *H*. *pylori* antibody test were collected for other assays. After a medical record review, individuals who were diagnosed with diabetes, hypertension, dyslipidemia were excluded, and those who smoke, take routine medication, or had past cancer history were excluded. The CLSI guideline recommends at least twenty samples to be tested to validate the manufacturer’s claimed reference interval. The reference intervals were considered valid if no more than two out of twenty reference values fall outside the limit.

### 2.2 Statistical analysis

The analysis of performance evaluation data was carried out using Microsoft Excel 2013 (Microsoft Corporation, WA, USA) for precision and carry-over rate, EP Evaluator 11 (Data Innovations, VT, USA) for linearity, AMR, and method comparison tests.

## 3 Results

### 3.1 Precision

%CVs and 95% CIs of repeatability (within-run precision) and within-laboratory precision were calculated (Table 1). Within-run %CVs ranged from 0.82 to 6.91%. The manufacturer’s specifications were observed to be exceeded in low-level testosterone, medium-level hsTnI, and high-level homocysteine. However, all within-laboratory %CVs met the manufacturer’s claimed precision, ranging from 1.00% to 7.84%.

**Table 1:** Repeatability and within-laboratory imprecision of the Alinity i system for 31 assays.

Assay	Unit	Testing Material	Level	Mean concentration	Repeatability	Within-laboratory precision
					%CV (95% CI)	%CV (95% CI)
TSH	uIU/mL	Abbott control	1	0.0934	1.30 (0.99–1.87)	1.55 (1.21–2.16)
			2	5.7528	1.26 (0.97–1.82)	1.26 (0.99–1.76)
			3	29.2811	1.78 (1.36–2.57)	1.99 (1.55–2.76)
TT3	ng/mL	Technopath IA Plus	1	95.96	1.48 (1.13–2.14)	1.58 (1.24–2.20)
			2	143.88	1.56 (1.19–2.25)	1.56 (1.22–2.18)
			3	287.94	3.01 (2.30–4.35)	3.31 (2.58–4.60)
FT3	pg/mL	Abbott control	1	2.92	2.97 (2.27–4.29)	2.97 (2.32–4.13)
			2	5.86	2.73 (2.09–3.94)	3.11 (2.43–4.33)
			3	10.01	1.97 (1.50–2.84)	1.97 (1.54–2.74)
TT4	ug/mL	Abbott control	1	4.46	2.17 (1.66–3.14)	2.17 (1.70–3.02)
			2	7.61	1.53 (1.17–2.20)	1.64 (1.28–2.28)
			3	15.11	2.17 (1.66–3.14)	2.59 (2.02–3.06)
FT4	ng/dL	Abbott control	1	0.58	3.31 (2.53–4.78)	3.31 (2.59–4.61)
			2	1.22	1.80 (1.37–2.59)	1.94 (1.51–2.70)
			3	2.83	4.00 (3.06–5.78)	4.00 (3.13–5.57)
Anti-TPO	IU/mL	Abbott control	1	0.63	2.82 (2.15–4.07)	2.84 (2.22–3.95)
			2	74.54	2.08 (1.59–3.01)	2.43 (1.90–3.38)
AFP	ng/mL	Abbott control	1	19.81	1.21 (0.93–1.75)	1.27 (0.99–1.77)
			2	195.20	1.87 (1.43–2.69)	1.87 (1.46–2.60)
			3	944.43	2.19 (1.67–3.16)	2.25 (1.76–3.13)
CEA	ng/mL	Abbott control	1	4.97	2.53 (1.93–3.65)	2.85 (2.22–3.96)
			2	19.89	1.94 (1.48–2.80)	2.60 (2.03–3.62)
			3	103.40	2.03 (1.55–2.93)	2.03 (1.58–2.82)
CA 19-9	U/mL	Abbott control	1	38.33	3.67 (2.81–5.30)	3.85 (3.01–5.36)
			2	144.75	4.41 (3.38–6.37)	4.85 (3.79–6.75)
			3	733.31	3.89 (2.97–5.62)	4.53 (3.54–6.30)
CA 125	U/mL	Abbott control	1	40.86	1.50 (1.15–2.17)	1.72 (1.34–2.39)
			2	297.93	2.06 (1.58–2.98)	2.32 (1.81–3.23)
			3	656.08	2.05 (1.57–2.96)	2.05 (1.60–2.85)
CA 15-3	U/mL	Abbott control	1	36.68	2.45 (1.87–3.54)	2.45 (1.91–3.41)
			2	253.45	2.60 (1.99–3.75)	2.82 (2.21–3.93)
HE 4	pmol/L	Abbott control	1	47.08	3.34 (2.56–4.82)	3.34 (2.61–4.55)
			2	166.82	2.89 (2.21–4.17)	2.89 (2.25–4.02)
			3	670.78	3.22 (2.47–4.66)	3.33 (2.60–4.63)
TPSA	ng/mL	Abbott control	1	0.504	1.97 (1.51–2.85)	2.34 (1.83–3.26)
			2	4.064	3.26 (2.49–4.70)	3.31 (2.58–4.60)
			3	23.779	3.40 (2.60–4.91)	3.57 (2.79–4.96)
FPSA	ng/mL	Abbott control	1	0.415	1.96 (1.50–2.83)	2.12 (1.66–2.95)
			2	1.011	2.03 (1.55–2.93)	2.28 (1.78–3.17)
			3	6.959	2.35 (1.80–3.39)	2.36 (1.84–3.28)
Testosterone	ng/mL	Abbott control	1	0.08	6.04^a^ (4.62–8.72)	6.04 (4.72–8.40)
			2	0.69	2.14 (1.64–3.09)	2.59 (2.02–3.60)
			3	2.32	1.49 (1.14–2.16)	1.64 (1.28–2.28)
Progesterone	ng/mL	Abbott control	1	0.87	5.13 (3.92–7.41)	5.28 (4.12–7.35)
			2	4.90	2.40 (1.83–3.46)	2.40 (1.87–3.33)
			3	20.91	2.38 (1.82–3.44)	2.39 (1.87–3.33)
E2	pg/mL	Abbott control	1	43.72	4.88 (3.74–7.05)	5.38 (4.20–7.49)
			2	180.24	2.39 (1.83–3.46)	3.62 (2.83–5.04)
			3	578.16	1.99 (1.52–2.87)	2.06 (1.61–2.87)
Prolactin	ng/mL	Abbott control	1	8.10	1.96 (1.50–2.82)	2.06 (1.61–2.86)
			2	20.94	1.46 (1.12–2.12)	1.60 (1.25–2.23)
			3	41.93	1.58 (1.21–2.28)	1.58 (1.24–2.20)
b-HCG	mIU/mL	Abbott control	1	25.13	2.88 (2.20–4.16)	2.90 (2.26–4.03)
			2	728.48	1.08 (0.82–1.55)	1.11 (0.87–1.55)
			3	4935.58	1.21 (0.92–1.74)	1.21 (0.94–1.68)
FSH	mIU/mL	Abbott control	1	4.91	1.78 (1.36–2.57)	1.87 (1.46–2.60)
			2	25.62	1.85 (1.42–2.67)	2.10 (1.64–2.92)
			3	77.53	2.40 (1.83–3.46)	2.70 (2.11–3.76)
LH	mIU/mL	Technopath IA Plus	1	3.78	1.94 (1.49–2.80)	1.94 (1.52–2.70)
			2	21.49	1.68 (1.29–2.43)	1.68 (1.31–2.34)
			3	43.67	1.19 (0.91–1.72)	1.39 (1.08–1.93)
Cortisol	ug/dL	Technopath IA Plus	1	3.83	3.24 (2.48–4.68)	3.24 (2.53–4.51)
			2	15.27	0.97 (0.74–1.40)	1.16 (0.90–1.61)
			3	32.76	1.30 (1.00–1.88)	1.42 (1.11–1.98)
hsTnI	ng/mL	Abbott control	1	0.019	3.54 (2.71–5.11)	3.60 (2.81–5.01)
			2	0.196	2.16^a^ (1.65–3.12)	2.30 (1.80–3.21)
			3	15.184	1.55 (1.18–2.24)	2.22 (1.73–3.09)
BNP	pg/mL	Abbott control	1	88.15	2.76 (2.11–3.99)	3.80 (2.96–5.28)
			2	493.60	2.49 (1.90–3.59)	3.07 (2.40–4.27)
			3	3416.88	0.82 (0.63–1.18)	1.00 (0.78–1.39)
Ferritin	ng/mL	Abbott control	1	20.58	2.95 (2.26–4.26)	2.96 (2.31–4.12)
			2	141.59	2.21 (1.69–3.19)	2.37 (1.85–3.30)
			3	380.22	2.41 (1.85–3.49)	2.41 (1.88–3.36)
iPTH	pg/mL	Abbott control	1	9.78	2.61 (2.00–3.77)	2.78 (2.17–3.87)
			2	66.13	2.12 (1.62–3.06)	2.28 (1.78–3.17)
			3	270.83	1.91 (1.46–2.76)	1.93 (1.51–2.69)
Folate	ng/mL	Abbott control	1	3.94	3.46 (2.65–4.99)	3.79 (2.96–5.27)
			2	7.40	3.14 (2.41–4.54)	3.14 (2.46–4.37)
			3	16.06	2.46 (1.88–3.55)	2.54 (1.99–3.54)
Vitamin B12	pg/mL	Abbot control	1	260.96	4.75 (3.63–6.86)	4.96 (3.87–6.89)
			2	476.04	3.46 (2.65–5.00)	4.26 (3.33–5.93)
			3	976.52	3.26 (2.49–4.70)	3.26 (2.54–4.53)
Vitamin D	ng/mL	Abbott control	1	20.34	2.21 (1.69–3.19)	2.86 (2.23–3.98)
			2	40.32	2.11 (1.62–3.05)	2.92 (2.28–4.07)
			3	76.02	1.18 (0.90–1.70)	2.38 (1.86–3.31)
C-peptide	ng/mL	Abbott control	1	0.96	2.88 (2.20–4.16)	2.88 (2.25–4.00)
			2	3.78	2.36 (1.81–3.41)	2.36 (1.84–3.29)
			3	16.55	1.11 (0.85–1.61)	1.41 (1.10–1.97)
Homocysteine	umol/L	Abbott control	1	7.47	2.16 (1.66–3.12)	2.17 (1.70–3.02)
			2	13.03	1.88 (1.44–2.71)	2.86 (2.24–3.98)
			3	25.43	1.86^a^ (1.43–2.69)	3.43 (2.67–4.77)

When within-run imprecision was larger than within-laboratory imprecision, within-run variance had been adopted as within-laboratory variance.

a Values exceed manufacturer’s imprecision specification.

### 3.2 Linearity and AMR

Linear regression was revealed to the best-fit model for TPSA, progesterone, E2, FSH, and homocystein. Recovery of these measurands was from 97.8 to 105.2% (Table 2). Polynomial regression was optimal for the other assays; the second-order regression for TT4, CEA, CA 19–9, HE 4, FPSA, prolactin, hsTnI, vitamin B12, 25-OH vitamin D, and C-peptide, and the third-order regression for the rest of assays with recovery distributed from 90.1 to 109.7%. When the values estimated from the best-fit polynomial regression were compared to those from linear regression, the nonlinearity ranged from −7.3 to 8.9%. Although the lowest concentration level of cortisol had exceeded the acceptable nonlinearity limit ±10%, the recovery of cortisol was within range of 96.3 and 101.6% for all concentration levels, and results from four replicates of level 1 (1.1 ㎍/dL) ranged from 1.0 to 1.2 ㎍/dL with imprecision from −9.1 to +9.1%. The high nonlinearity of polynomial regression in low-level specimen seems due to growing effect of y-intercept at this point. The coefficients of determination (R^2^) for all thirty-one assays evaluated in this study were larger than 0.99.

**Table 2:** Linearity and AMR validation results of the Alinity i system.

Assay	Unit	Testing Material	Manufacture’s claimed AMR		Validated AMR		Best fit	Non-linearity (%)	Recovery (%)
			Low	High	Low	High			
TSH	uIU/mL	Serum	0.0083	100	0.0207	85.2935	3rd order	−3.8	100.0–105.5
TT3	ng/mL	Validate	0.4	6.0	0.43	5.80	3rd order	−5.1	93.9–100.3
FT3	pg/mL	Validate	1.5	20.0	0.78	19.94	3rd order	−5.2	93.0–102.3
TT4	ug/dL	Calibrator	3.0	24.0	3.21	23.68	2nd order	5.9	94.1–100.0
FT4	ng/dL	Validate	0.42	5.0	0.43	4.28	3rd order	3.6	97.0–104.3
Anti-TPO	IU/mL	Serum	3.0	1000	3.68	977.91	3rd order	−2.6	97.0–100.0
AFP	ng/mL	Serum	2	2000	2.16	1641.21	3rd order	4.3	92.9–100.0
CEA	ng/mL	Validate	1.73	1500	2.09	1266.41	2nd order	−2.5	95.5–100.0
CA 19-9	U/mL	Validate	2.06	1200	1.24	1195.19	2nd order	−5.7	93.1–100.0
CA 125	U/mL	Calibrator	1.1	1000	1.00	988.9	3rd order	−2.8	100.0–106.2
CA 15-3	U/mL	Serum	0.6	800	0.65	682.23	3rd order	5.6	93.0–100.0
HE 4	pmol/L	Calibrator	20	1500	2.33	1337.25	2nd order	−3.2	100.0–106.0
TPSA	ng/mL	Validate	0.025	100	0.029	79.339	Linear	N/A	100.0–104.5
FPSA	ng/mL	Validate	0.021	30	0.023	26.810	2nd order	−1.4	98.3–100.9
Testosterone	ng/mL	Validate	0.04	18.62	0.19	15.31	3rd order	−2.4	96.4–101.0
Progesterone	ng/mL	Validate	0.5	40	0.30	33.50	Linear	N/A	98.8–100.0
E2	pg/mL	Serum	24	1000	24.0	1000.0	Linear	N/A	100.0–108.8
Prolactin	ng/mL	Validate	0.82	200	0.68	199.97	2nd order	−7.3	91.7–100.0
b-HCG	mIU/mL	Calibrator	2.3	15,000	2.22	14509.89	3rd order	−2.4	97.8–102.7
FSH	ng/mL	Validate	0.11	150.0	0.05	130.88	Linear	N/A	99.2–100.5
LH	mIU/mL	Calibrator	0.12	250	0.48	247.88	3rd order	6.7	98.1–109.0
Cortisol	ug/dL	Serum	1.0	59.8	1.10	44.10	3rd order	24.1^a^	96.3–101.6
hsTnI	pg/mL	Calibrator	10	50,000	1.48	46497.08	2nd order	5.0	100.0–106.5
BNP	pg/mL	Calibrator	10	5000	6.00	4999.73	3rd order	7.9	98.5–108.6
Ferritin	ng/mL	Validate	1.98	1675.56	1.78	1605.82	3rd order	6.2	93.9–107.9
iPTH	pg/mL	Calibrator	3.0	3000	3.73	2691.70	3rd order	8.9	100.0–109.7
Folate	ng/mL	Validate	2.2	20	1.98	14.08	3rd order	5.9	92.8–101.5
Vitamin B12	pg/mL	Validate	148	2000	154.50	1954.00	2nd order	−6.2	97.5–108.7
Vitamin D	ng/mL	Calibrator	3.5	154.2	3.15	111.90	2nd order	−7.3	90.1–100.0
C-peptide	ng/mL	Serum	0.03	30	0.18	36.93	2nd order	−1.6	100.0–102.8
Homocyteine	umol/L	Serum	0.1	50	1.3	35.10	Linear	N/A	97.8–100.0

a Materials which have showed non-linearity larger than 10% at the lowest concentration. However, the recovery was within acceptable limit for all concentration levels.

### 3.3 Method comparison

**Fig. 1:**
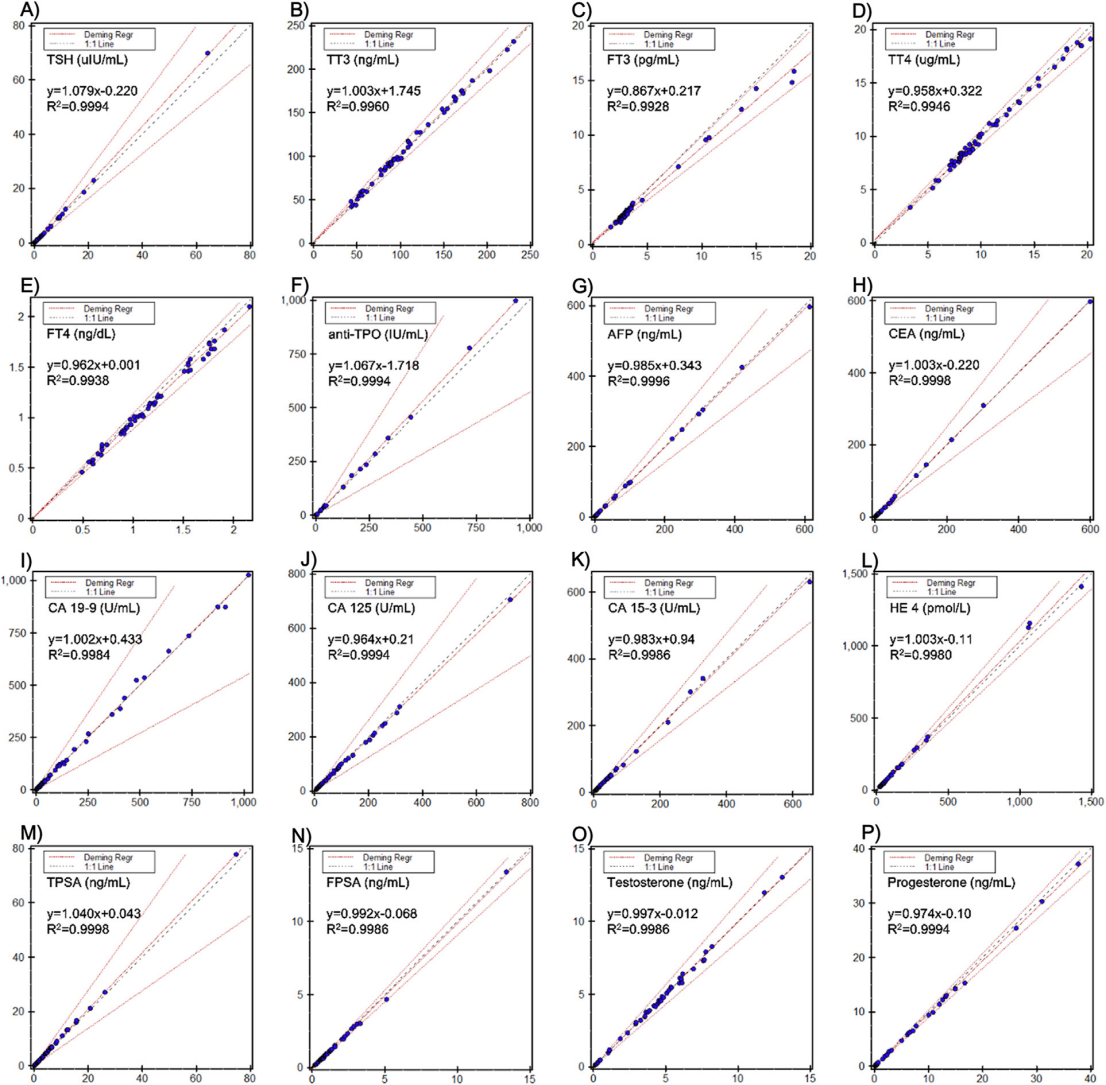

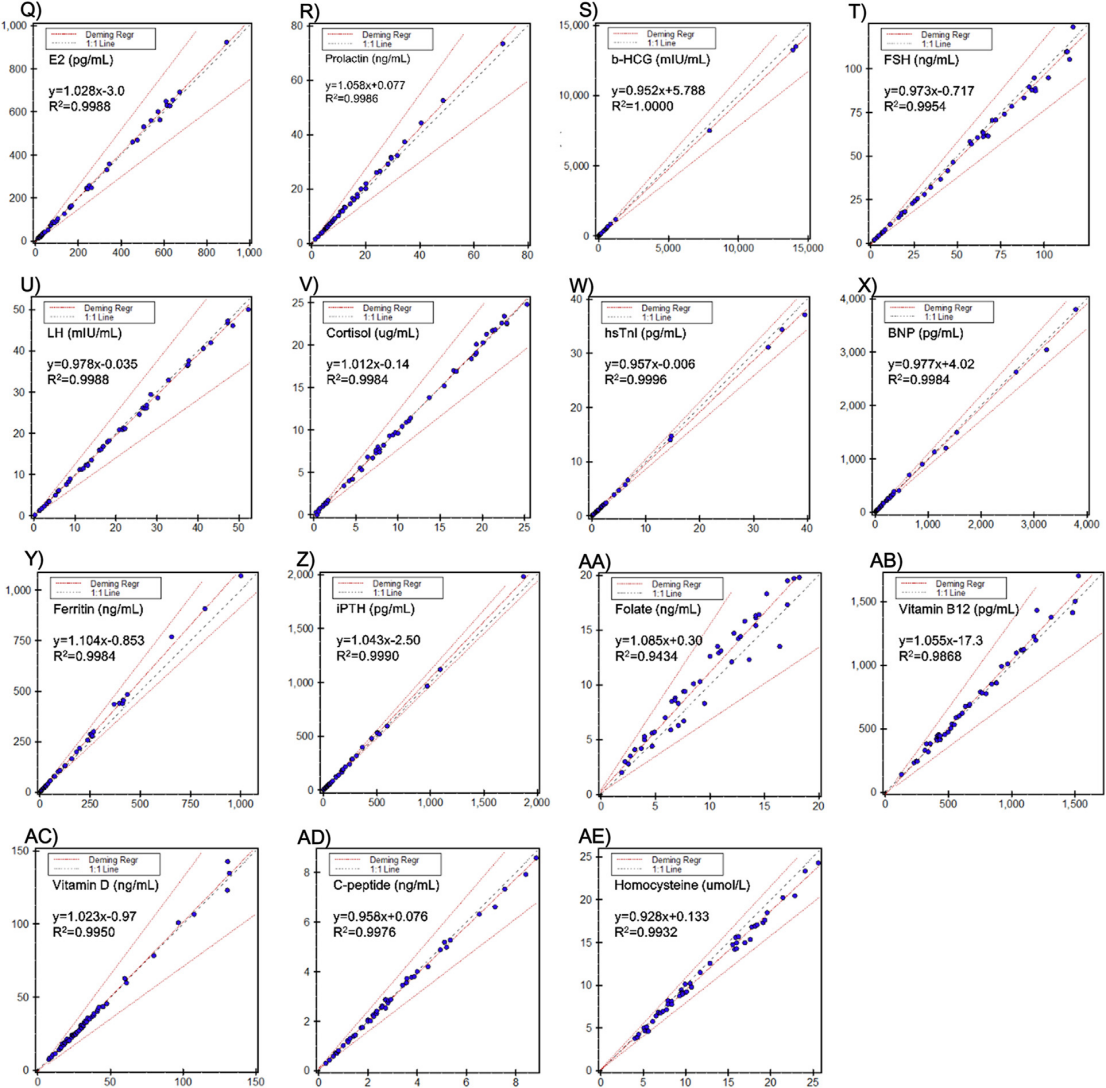
The results of method comparison between Alinity i and Architect i2000sr system for thirty-one assays. A)-F) TSH, TT3, FT3, TT4, FT4, and anti-TPO; G)-N) AFP, CEA, CA 19–9, CA 125, CA 15–3, HE 4, TPSA, and FPSA; O)–V) testosterone, progesterone, E2, prolactin, b-HCG, FSH, LH, and cortisol; W)-X) hsTnI, and BNP; Y)-AE) ferritin, iPTH, folate, vitamin B12, 25-OH vitamin D, C-peptide, and homocysteine. The test results of each sample were presented as dot, results from Alinity i system on Y-axis, whereas those from Architect i2000sr on X-axis. The dot-and-dash lines represent Deming regression and allowable percent difference, and diagonal dash line represents identity line.

The results from the Alinity i were well correlated with the Architect i2000sr (Fig. 1). There was no statistical difference between two systems for TT3, CA 19–9, CEA, and testosterone by Deming regression, with 95% CI of a slope and an intercept containing 1 and 0, respectively. For the other assays, there was statistical difference with the mean %bias smaller than the allowable limit and without any clinical significance (Table 3).

**Table 3:** Method comparison between the Alinity i system and Architect i2000sr.

Assay	Unit	n	Deming		R^2^	TEa (%)	vs. Architect	
			Slope (95% CI)	Intercept (95%CI)			Mean %bias	Decision
TSH	uIU/mL	50	1.079 (1.071, 1.087)	−0.220 (−0.307, −0.133)	0.9994	23.7	2.75	Equivalent
TT3	ng/mL	50	1.003 (0.985, 1.022)	1.745 (−0.411, 3.900)	0.9960	9.22	1.94	Equivalent
FT3	pg/mL	60	0.867 (0.847, 0.886)	0.217 (0.109, 0.325)	0.9928	11.3	−8.09	Equivalent
TT4	ug/mL	60	0.958 (0.940, 0.977)	0.322 (0.115, 0.529)	0.9946	7.0	−1.09	Equivalent
FT4	ng/dL	50	0.962 (0.940, 0.984)	0.001 (−0.026, 0.028)	0.9938	8.0	−3.84	Equivalent
Anti-TPO	IU/mL	50	1.067 (1.059, 1.074)	−1.718 (−3.178, −0.259)	0.9994	46.2	4.37	Equivalent
AFP	ng/mL	50	0.985 (0.980, 0.990)	0.343 (−0.318, 1.005)	0.9996	21.9	−0.86	Equivalent
CEA	ng/mL	50	1.003 (0.999, 1.007)	−0.220 (−0.634, 0.194)	0.9998	24.7	−0.27	Equivalent
CA 19-9	U/mL	50	1.002 (0.990, 1.013)	0.433 (−3.141, 4.007)	0.9984	46.03	0.42	Equivalent
CA 125	U/mL	50	0.964 (0.958, 0.971)	0.21 (−0.75, 1.18)	0.9994	35.4	−3.40	Equivalent
CA 15-3	U/mL	50	0.983 (0.972, 0.993)	0.94 (−0.38, 2.26)	0.9986	20.8	−0.04	Equivalent
HE 4	pmol/L	50	1.033 (1.019, 1.047)	−0.11 (−4.42, 4.20)	0.9980	10.0^a^	3.19	Equivalent
TPSA	ng/mL	50	1.040 (1.035, 1.044)	0.043 (−0.016, 0.102)	0.9998	33.6	4.52	Equivalent
FPSA	ng/mL	54	0.992 (0.982, 1.003)	−0.068 (−0.092, −0.044)	0.9986	8.0^a^	−5.91	Equivalent
Testosterone	ng/mL	50	0.997 (0.987, 1.008)	−0.012 (−0.064, 0.040)	0.9986	13.61	−0.58	Equivalent
Progesterone	ng/mL	50	0.974 (0.967, 0.981)	−0.10 (−0.16, −0.03)	0.9994	7.0^a^	−4.46	Equivalent
E2	pg/mL	50	1.028 (1.017, 1.038)	−3.0 (−6.4, 0.4)	0.9988	26.86	1.40	Equivalent
Prolactin	ng/mL	50	1.058 (1.046, 1.069)	0.077 (−0.162, 0.316)	0.9986	29.4	6.08	Equivalent
b-HCG	mIU/mL	52	0.952 (0.951, 0.954)	5.788 (1.100, 10.477)	1.0000	10.0^a^	−4.16	Equivalent
FSH	ng/mL	50	0.973 (0.954, 0.993)	−0.717 (−1.914, 0.479)	0.9954	21.19	−4.22	Equivalent
LH	mIU/mL	50	0.978 (0.968, 0.988)	−0.035 (−0.271, 0.201)	0.9988	27.92	−2.40	Equivalent
Cortisol	ug/dL	50	1.012 (1.001, 1.023)	−0.14 (−0.29, 0.00)	0.9984	22.8	−0.19	Equivalent
hsTnI	pg/mL	50	0.957 (0.951, 0.963)	−0.006 (−0.060, 0.049)	0.9996	10.0^a^	−4.55	Equivalent
BNP	pg/mL	50	0.977 (0.966, 0.989)	4.02 (−5.92, 13.97)	0.9984	12.0^a^	−1.27	Equivalent
Ferritin	ng/mL	50	1.104 (1.091, 1.117)	−0.853 (−4.573, 2.866)	0.9984	16.9	9.43	Equivalent
iPTH	pg/mL	60	1.043 (1.035, 1.052)	−2.50 (−5.62, 0.63)	0.9990	7.0^a^	2.91	Equivalent
Folate	ng/mL	50	1.085 (1.004, 1.166)	0.30 (−0.55, 1.15)	0.9434	39.0	11.08	Equivalent
Vitamin B12	pg/mL	50	1.055 (1.019, 1.091)	−17.3 (−45.4, 10.9)	0.9868	30.0	3.0	Equivalent
Vitamin D	ng/mL	60	1.023 (1.004–1.042)	−0.97 (−1.86, −0.09)	0.9950	30.0	−0.34	Equivalent
C-peptide	ng/mL	50	0.958 (0.945, 0.972)	0.076 (0.028, 0.125)	0.9976	20.8	−1.57	Equivalent
Homocysteine	umol/L	50	0.928 (0.905, 0.950)	0.133 (−0.164, 0.430)	0.9932	15.48	−6.31	Equivalent

Regression equations were calculated assuming the results from the Alinity i system as Y method and those from the Architect i2000sr as X method..

a Manufacturer’s claimed values were adopted as total allowable error because the Westgard desirable biological variation database did not clarify the values for these analytes.

### 3.4 Carry-over rate

Carry-over rates ranged between 0 and 0.89% and no significant carry-over was observed. (Data not shown).

### 3.5 Reference interval validation

The reference intervals were validated with no more than 2 out of twenty measured reference values for all assays falling outside those claimed by the manufacture (Table 4).

**Table 4:** Validation of the Reference intervals.

Assay	Subgroup	n	Unit	Manufacturer-reported reference intervals		Number of samples falling outside the interval	Decision
				Lower limit	Upper limit		
TSH		20	uIU/mL	0.35	4.94	0	Validated
TT3		20	ng/mL	64	152	0	Validated
FT3		20	pg/mL	1.88	3.18	2	Validated
TT4		20	ug/dL	4.87	11.72	2	Validated
FT4		20	ng/dL	0.70	1.48	0	Validated
Anti-TPO		20	IU/mL		5.61	0	Validated
AFP		20	ng/mL		8.78	0	Validated
CEA		20	ng/mL		5.0	0	Validated
CA 19-9		20	U/mL		37.0	0	Validated
CA 125		20	U/mL		35.0	0	Validated
CA 15-3		20	U/mL		31.3	0	Validated
TPSA	Male only	20	ng/mL		4.0	0	Validated
Testosterone	Male, age<50	20	ng/mL	2.40	8.71	2	Validated
	Male, age≥50	20		2.21	7.16	2	Validated
	Female, age<50	20		0.14	0.53	0	Validated
	Female, age≥50	20		0.12	0.36	2	Validated
Prolactin	Male	20	ng/mL	3.46	19.40	0	Validated
	Female	20		5.18	26.53	2	Validated
b-HCG		20	mIU/mL		5.0	1	Validated
hsTnI		20	pg/mL		0.0262	1	Validated
BNP		20	pg/mL		100^a^	0	Validated
Ferritin	Male	20	ng/mL	21.81	274.66	2	Validated
	Female	20		4.63	204.0	0	Validated
iPTH		20	pg/mL	15.0	68.3	2	Validated
Folate		20	ng/mL	3.1	20.5	1	Validated
Vitamin B12		20	pg/mL	187	883	2	Validated
C-peptide		20	ng/mL	0.78	5.19	1	Validated
Homocyteine		20	umol/L	5.08	15.39	1	Validated

a The FDA-approved cutoff for BNP was adopted as the upper limit of reference, which was also suggested as a decision threshold by the manufacturer, due to the characteristic of the analyte of dynamic changes by ages and severity of cardiac failure.

## 4 Discussion

Immunoassays are analytical methods that utilize the reaction between antigen and antibody in the quantification of analytes. This method has high sensitivity and specificity, high throughput, and applicable for a wide range of analytes which are difficult to measure using other analytical methods [8]. Therefore, it has been widely used to measure drug concentration, therapeutic drug monitoring, hormones, and diseases-specific proteins including cardiac injury markers, and tumor markers [[14], [15], [16]].

When the newly developed instrument is introduced to laboratories to replace existing analyzer for routine clinical tests, analytical performance should be evaluated whether it can provide reliable test results that the laboratory requires. In this study, the authors have evaluated the analytical performance of Alinity i system, the novel analytical platform, in terms of precision, linearity and AMR, correlation with Architect i2000sr analyzer, and carry-over rate.

As a result, the Alinity i system revealed to have acceptable analytical performance. Precision of the assays was excellent with within-laboratory %CVs within the manufacturer’s specification for all assays with all levels of concentration. In linearity evaluation, all the assays have met the acceptable linearity criteria, best-fit first order regression for TPSA, progesterone, E2, FSH, and homocystein and the best-fit with nonlinearity smaller than 10% for the other assays by polynomial regression. The coefficients of determination (R^2^) were lager than 0.99 for all assays. When compared to the results obtained from Architect i2000sr analyzer, the two instruments were highly correlated with the coefficients of determination (R^2^) larger than 0.95 for most assays except for folate with R^2^ slightly smaller than 0.95. However, it was clinically insignificant with the mean percent bias smaller than desirable total allowable error (TEa) suggested by the Westgard database. Carry-over rates were smaller than 1.0% for all assays.

The manufacturer’s specifications were not met for repeatability of some assays, while it was observed to be fit in the previous study by the manufacturer’s initiative study [17]. This result seems to be mainly originated from the difference between the highly controlled manufacturer’s facility and a routine clinical laboratory. In addition, the entire manufacturer’s claimed AMR were not covered for some assays in linearity tests, which leads to a careful interpretation of test results that may be produced out of the verified ranges in clinical practice. And there were some low-level samples with bias exceeding the Westgard TEa by interpolation of the Deming regression equation. This might be due to the growing systematic bias of regression with low concentrations and influence of y-intercept.

Despite a number of immunoassays was evaluated based on the internationally recognized method, this study still has some limitations. There was lack of test samples near AMR concentration at upper and lower limit in method comparison. The test affecting factors for menstrual cycle in FSH and LH and circadian cycle in 25-OH vitamin D could not be controlled, resulting in exclusion for RI validation. Additionally, as a single center study, potential imprecision that can arise from inter-laboratory difference in multicenter study was not included in this study also.

In conclusion, the Alinity i system showed good analytical performance in precision, linearity and AMR, carry-over, correlation and agreement with the established Architect i2000sr system. It would have added more value if inter-laboratory difference was included in method comparison. As a result, this new analytical system is expected to replace the current system and be readily utilized for clinical use in laboratories with advanced processing speed and throughput.

## Funding

This study was supported by 10.13039/100014386Abbott Diagnostics Korea.

## CRediT authorship contribution statement

**Jong Do Seo:** Conceptualization, Formal analysis, Writing - original draft, The authors certified that each author participated sufficiently in the study conception or design, data analysis or interpretation, and drafting or revision of the manuscript, so that each author takes responsibility for the validity and objectivity of the entire study. And each author has approved the final version of the manuscript. Neither this manuscript nor one with similar content has been published or is being considered for publication in any language, except as an abstract or academic thesis. **Da Young Song:** Conceptualization, Formal analysis, Writing - original draft, The authors certified that each author participated sufficiently in the study conception or design, data analysis or interpretation, and drafting or revision of the manuscript, so that each author takes responsibility for the validity and objectivity of the entire study. And each author has approved the final version of the manuscript. Neither this manuscript nor one with similar content has been published or is being considered for publication in any language, except as an abstract or academic thesis. **Youngwon Nam:** Conceptualization, Formal analysis, Writing - original draft, The authors certified that each author participated sufficiently in the study conception or design, data analysis or interpretation, and drafting or revision of the manuscript, so that each author takes responsibility for the validity and objectivity of the entire study. And each author has approved the final version of the manuscript. Neither this manuscript nor one with similar content has been published or is being considered for publication in any language, except as an abstract or academic thesis. **Chihchiao Li:** Conceptualization, Formal analysis, Writing - original draft, The authors certified that each author participated sufficiently in the study conception or design, data analysis or interpretation, and drafting or revision of the manuscript, so that each author takes responsibility for the validity and objectivity of the entire study. And each author has approved the final version of the manuscript. Neither this manuscript nor one with similar content has been published or is being considered for publication in any language, except as an abstract or academic thesis. **Seunghwan Kim:** Conceptualization, Formal analysis, Writing - original draft, The authors certified that each author participated sufficiently in the study conception or design, data analysis or interpretation, and drafting or revision of the manuscript, so that each author takes responsibility for the validity and objectivity of the entire study. And each author has approved the final version of the manuscript. Neither this manuscript nor one with similar content has been published or is being considered for publication in any language, except as an abstract or academic thesis. **Joon Hee Lee:** Conceptualization, Formal analysis, Writing - original draft, The authors certified that each author participated sufficiently in the study conception or design, data analysis or interpretation, and drafting or revision of the manuscript, so that each author takes responsibility for the validity and objectivity of the entire study. And each author has approved the final version of the manuscript. Neither this manuscript nor one with similar content has been published or is being considered for publication in any language, except as an abstract or academic thesis. **Kyunghoon Lee:** Conceptualization, Formal analysis, Writing - original draft, The authors certified that each author participated sufficiently in the study conception or design, data analysis or interpretation, and drafting or revision of the manuscript, so that each author takes responsibility for the validity and objectivity of the entire study. And each author has approved the final version of the manuscript. Neither this manuscript nor one with similar content has been published or is being considered for publication in any language, except as an abstract or academic thesis. **Junghan Song:** Conceptualization, Formal analysis, Writing - original draft, The authors certified that each author participated sufficiently in the study conception or design, data analysis or interpretation, and drafting or revision of the manuscript, so that each author takes responsibility for the validity and objectivity of the entire study. And each author has approved the final version of the manuscript. Neither this manuscript nor one with similar content has been published or is being considered for publication in any language, except as an abstract or academic thesis. **Sang Hoon Song:** Conceptualization, Formal analysis, Writing - original draft, The authors certified that each author participated sufficiently in the study conception or design, data analysis or interpretation, and drafting or revision of the manuscript, so that each author takes responsibility for the validity and objectivity of the entire study. And each author has approved the final version of the manuscript. Neither this manuscript nor one with similar content has been published or is being considered for publication in any language, except as an abstract or academic thesis.

## Declaration of competing interest

The authors declared no potential conflicts of interest with respect to the research, authorship, and/or publication of this article.
